# Editorial: Spaces for sustainable food systems and healthy diets

**DOI:** 10.3389/fnut.2026.1932612

**Published:** 2026-08-05

**Authors:** Sebastian Elgueta, Leslie Landaeta-Diaz

**Affiliations:** 1 Facultad de Ciencias de la Rehabilitación y Calidad de Vida, Escuela de Nutrición y Dietética, Universidad San Sebastián, Providencia, Chile; 2 Núcleo de Investigación en Nutrición y Ciencias Alimentarias, Universidad de Las Américas, Santiago, Chile

**Keywords:** food environments, food policy, nutrition transition, planetary health diets, sustainable food systems

The food system plays a crucial role in shaping both human health and the health of our planet. In 2018, dietary risks contributed to 11 million deaths, about 22% of all adult deaths, including 10 million from cardiovascular issues (1). Food system emissions reached 18 gigatons of CO_2_ equivalent in 2015, accounting for roughly 34% of global greenhouse gases, with 71% originating from agriculture and land use (2). Over one billion people are living with obesity today, approximately 879 million adults and 159 million children and adolescents (3), while around 673 million suffered from hunger in 2024 (4). By 2050, the world's population is expected to reach about 9.7 billion (5), further increasing food demand. The EAT-Lancet Commission proposes that shifting toward plant-based diets, reducing food waste, and improving production practices could help are essential to nourish this growing population within planetary boundaries (6). This framework has shaped debate, and its target's feasibility remains under discussion.

Space is a critical factor in addressing this previous challenge: the locations where food is cultivated, marketed, and consumed play a decisive role in determining access to a nutritious diet. Food environments serve as the interface through which individuals interact with the broader food system, mirroring aspects such as urban planning, infrastructure, and policy (7). This Research Topic asked how spatial organization, governance, and consumer behavior move food systems toward health and sustainability. We gathered 11 articles across several regions and disciplines. This editorial groups them under four areas.

The first focuses on assessing and reshaping the food environment. Sun et al. examined neighborhoods in Tianjin, China, and used both objective data and residents' perceptions. They identified only a few traditional food deserts, and that neighborhoods with many supermarkets and fresh markets were associated with higher obesity rates, challenging Western assumptions when applied to a Chinese context. Xie examined the United States Food Access Research Atlas in Durham, North Carolina. Larger stores provided healthier and more affordable food than the Atlas suggested, so equating a lack of supermarkets with a food desert can be misleading for policy decisions. Maddock et al. outline a plan to study residents of an agrihood, a community designed around a working farm, and will monitor their diet, activity levels, and cardiometabolic health over time. These studies collectively suggest that spatial metrics need to be tailored to the local context.

The second area covers diet quality and the planetary health diet. Grant et al. mapped adherence to the planetary health diet across 11 European countries in the PLANET project. Adherence was low everywhere, though higher in Southern Europe and among women, and their WISH 2.0 index tracked real consumption better than existing tools. Priulli et al. examined children in Vicosa, Brazil, and observed that those from poorer, food-insecure households tended to have diets that matched the typical patterns, which appeared to reflect financial constraints rather than personal preferences. Jia and Xue examined research on the Mediterranean diet in China from 2006 to 2025, highlighting a shift toward culturally tailored, digitally supported, and climate-conscious models such as the Jiangan diet. These studies emphasize that global dietary guidelines must be adapted locally to be effective.

The third area addresses communication and consumer behavior, including tools for evaluating sustainable options. Berardy et al. developed a color-coded matrix that presents the carbon footprint and health effects of 30 food groups in a single figure. The tool favors plant-based, less-processed foods and guides clearer choices. Liu et al. developed a scale to assess how Chinese consumers perceive the benefits of insect-based foods, highlighting aspects such as health, nutrition, taste, culture, cost, and environmental impact. These studies suggest practical ways to make sustainable choices more visible and appealing.

The fourth area focuses on production and structural change. Jin and Zhu studied rural population aging and land ecological security in China. They found that aging was associated with lower land ecological security, but farm services, land transfers, and larger-scale operations helped mitigate some of the damage. Zheng et al. analyzed Chinese household panel data and found that owning a refrigerator was associated with a 39% higher daily calorie intake and a shift toward higher-fat and processed foods. This illustrates a connection between food safety technology and rising obesity risk during the nutrition transition, as discussed by Popkin et al. (8).

Most contributions originate from China, Europe, the United States, and Brazil, reflecting both widespread interest and highlighting a gap in coverage of Africa and low-income regions. Many studies question ideas developed in high-income countries when applied elsewhere, addressing issues from food deserts to global diet indices. Others suggest that structural factors such as income, refrigeration, and store networks influence diet more than individual choices. These studies also emphasize spatial and equity considerations, exploring what constitutes a healthy diet, where it is accessible, and for whom.

Most designs are cross-sectional, which limits our ability to draw causal conclusions. However, the agrihood protocol and aging analysis are beginning to examine how things change over time, and more long-term studies are needed. We still have limited evidence from Africa, South Asia, and informal food markets. Future research should explore spatial interventions, adapt diet metrics to fit different cultural contexts, and examine how changes in governance affect vulnerable groups. Policymakers should see proximity, affordability, and cultural importance as interconnected issues rather than separate ones.

The published articles suggest that sustainable food systems and healthy diets rely on proper space, structure, and fairness. We appreciate the efforts of the authors, reviewers, and the Frontiers team. We hope this Research Topic supports researchers and decision-makers in creating food environments that benefit both people and the planet.
